# Quality of life and paracetamol in advanced dementia (Q-PID): protocol of a randomised double-blind placebo-controlled crossover trial

**DOI:** 10.1186/s12877-018-0974-1

**Published:** 2018-11-14

**Authors:** Paulien H. van Dam, Wilco P. Achterberg, Jacobijn Gussekloo, Bettina S. Husebo, Monique A. A. Caljouw

**Affiliations:** 1 Department of Public Health and Primary Care, Leiden University Medical Center, P.O. Box 9600, 2300 RC, Leiden, The Netherlands; 2 Section of Gerontology and Geriatrics, Department of Internal Medicine, Leiden University Medical Center, Leiden, The Netherlands; 3 Department of Global Public Health and Primary Care, Centre for Elderly – and Nursing Home Medicine, University of Bergen, Kalfarveien 31, N-5020 Bergen, Norway; 4 Municipality of Bergen, Bergen, Norway

**Keywords:** Quality of life, Paracetamol, Dementia, Nursing home, QUALIDEM

## Abstract

**Background:**

No proven effective interventions on quality of life (QoL) are available for persons with dementia in a long-term care facility (LTCF). However, several interventions are effective in diminishing mediators of QoL (i.e. challenging behaviour, depressed mood, sleeping disorders), including pain treatment. Un(der)diagnosed and un(der)treated pain is a serious and frequent problem in persons with dementia. Also, although pain is difficult to assess in this group, the impact on QoL is probably considerable. There is evidence that pain has a negative impact on behaviour, mood, functioning and social participation, and benefit may be derived from use of paracetamol. Therefore, in LTCF residents with advanced dementia, this study aims to evaluate the effect of scheduled pain treatment with paracetamol on QoL, neuropsychiatric symptoms, ADL function, pain, care dependency, and (change in) use of psychotropic and pain medication.

**Methods:**

This randomised, double-blind, placebo-controlled crossover trial will include 95 patients with: 1) age ≥ 65 years, 2) advanced dementia (Reisberg Global Deterioration Scale 5–7), and 3) QUALIDEM score ≤ 70. Exclusion criteria are the regular use of pain treatment, allergies to the study drugs, severe liver insufficiency or disease, use of > 4 units of alcohol/day, weight < 50 kg, and/or concomitant use of flucloxacillin. The two treatment periods of six weeks each (paracetamol and corresponding placebo) will be separated by a washout period of seven days. Primary outcome is effect on QoL (QUALIDEM and DS-DAT) and secondary outcome is effect on neuropsychiatric symptoms, ADL function, pain, care dependency, and (change in) use of psychotropic and pain medication (all compared to baseline).

**Discussion:**

If regular treatment with paracetamol proves to be beneficial for QoL, this could have major implications for daily practice in long-term care. Information from this study may help professionals in their decision making regarding the prescription of pain medication to improve the QoL of persons with dementia and a low QoL.

**Trial registration:**

The trial was registered on the Netherlands Trial Register (NTR6766); Trial registration date: 20th October, 2017.

## Background

The main goal of caring for persons with dementia living in long-term care facilities (LTCF) is the maintenance and/or improvement of their quality of life (QoL) [1]. QoL in persons with dementia involves multi-dimensional wellbeing on various domains, all influenced by the severity of dementia as well as individual and environmental factors. QoL can be affected by cognitive and functional decline, as well as by behavioural and psychological symptoms of dementia, and the quality of care received [2]. The LTCFs of the University Network of the Care sector South Holland (UNC-ZH) give the highest priority to the challenge of making an individual’s life with dementia bearable and to help achieve an optimal QoL.

Admission of a person with dementia to a LTCF is usually based on a combination of factors in many domains, in which care and treatment at home are insufficient to handle all the needs. The expected increase in the number of persons with dementia emphasises the need to cope with the difficulties that formal caregivers (elderly care physicians, nursing staff, paramedical staff) and informal caregivers (family/spouses) experience daily to maintain an optimal QoL in these individuals.

The appreciation and rating of an individual’s QoL is (conceptually) something that a person should report themselves. However, although some persons with dementia can give self-reported ratings in earlier stages of the disease, in more advanced stages this competency is often lost and assessment of QoL then generally relies on proxy observations.

The prevalence of pain in persons with dementia is high; it is reported that 40–60% of this group regularly experiences pain; for example pain is reported in 32, 43 and 57% of persons with dementia in Italy, the Netherlands and Finland, respectively [3]. Pain can have a negative influence on QoL in many ways; although pain is difficult to assess in persons with dementia, the impact on QoL is probably considerable. Studies have shown the beneficial effects of pain treatment in persons with dementia on outcomes other than the pain itself [4, 5]. Especially behavioural problems, sleeping and night-time behaviours, and social activities responded positively after active treatment with pain medication (irrespective of whether or not pain was present) [4–6]. In an earlier study, a secondary analysis suggested that pain management is also beneficial for mood (depression), apathy, staff distress, activities of daily living (ADL), appetite and eating disturbances [7, 8]; however, in this latter trial, the stepwise approach of treating pain was neither placebo-controlled nor blinded.

The complete mechanism of action of paracetamol, also known as acetaminophen, is still unclear [9]. Thus, the question remains whether paracetamol has only analgesic and antipyretic effects and, thereby, improves QoL, or whether it has another (yet unknown) independent mechanism of action on well-being that has not yet been revealed. Over all, the effects of regular pain treatment (not only with paracetamol) on QoL in persons with dementia have not yet been studied [4, 6, 10–12].

This proposed study will help gain more insight into and knowledge on the effect of pain treatment on QoL, neuropsychiatric symptoms, ADL function, pain, care dependency, and (change in) use of psychotropic and pain medication. Hopefully, the results will help persons with dementia to achieve and/or sustain the highest possible QoL, by alleviating undesired symptomatology.

### Aims of the Q-PID trial

The primary objective of the Q-PID trial is to evaluate the effect of scheduled administration of pain treatment with paracetamol on QoL of people with advanced dementia in LTCFs. Secondary aims are to evaluate the effects of regular pain treatment with paracetamol on neuropsychiatric symptoms, ADL function, pain, care dependency, and (change in) use of psychotropic and pain medication.

## Methods

### Design and study population

This 13-week double-blind, randomised, placebo-controlled crossover trial, is designed to include 95 residents with advanced dementia, being admitted to LTCFs affiliated with the UNC-ZH. Inclusion criteria are 1) age ≥ 65 years, 2) advanced dementia (Reisberg Global Deterioration Scale (GDS) 5–7 [13]) and 3) QUALIDEM score ≤ 70. This cut-off point is based on the median QUALIDEM total score that emerged from data of the STA-OP! study [14]. Exclusion criteria are the regular use of pain treatment [residents with paracetamol that is prescribed PRN (pro re nata, or ‘as needed’) are eligible only if the use of paracetamol in the previous week was ≤3 g/week with a maximum of 1 g/day], allergies to the study drugs (paracetamol or placebo), severe liver insufficiency or disease, use of > 4 units of alcohol/day, weight < 50 kg and/or concomitant use of flucloxacillin (because of possible interaction between paracetamol and flucloxacillin in women of advanced age, leading to an anion gap metabolic acidosis) [15].

### Recruitment and consent

Eligible residents and their legal representatives will be selected by the treating elderly care physician on the basis of not using any pain medication, or using paracetamol PRN ≤ 3 g/week with a maximum of 1 g/day, after which the legal representative receives the patient information letter that explains the purpose/procedures of the proposed study, the tests and questionnaires required, and possible hazards that might be involved. The legal representative is asked to return the consent form to the researchers by mail, either with a consent, or with refusal for the patient to participate. The patient information letter is re-send after non-response of the legal representative after four weeks. If the legal representative sends his/her consent, the researcher or research nurse contacts that legal representative by telephone to ensure that the right person has signed the form, and to answer any questions (if necessary). Once the researcher or research nurse has assured him/herself, then he/she also signs the consent form. A copy is added to the medical record of the patient in the nursing home. Thereafter, the resident is enrolled in the study. A case report form is kept of all participants in the study.

### Treatment

**Fig. 1:**
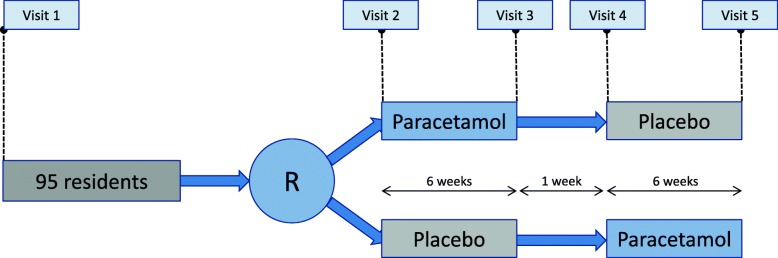
Flowchart of the Q-PID crossover trial. R = randomisation. Visit 1: Screening for inclusion and exclusion criteria. Visit 2: Baseline measurements. Visit 3 and 4: Follow-up measurements. Visit 5: Final and closing measurements

Participants receive orally administered paracetamol (or placebo, if they are randomised into starting placebo first) at a daily dose of 3 g for four weeks (3 × 2 tablets of 500 mg each), followed by administration of 2.5 g/day for two weeks, according to recent protocols of chronic use of paracetamol in older people [16]. After a wash-out period of seven days, a second six-week administration period starts with corresponding placebo (or paracetamol if the participant started with placebo) (see Fig. 1). The placebo tablets resemble the paracetamol tablets in colour, size and composition, and contain quinine to give a bitter taste. All study medication is packed in similar medication baskets.

Participants are allowed to use co-medication. If additional pain medication is needed during the study, administration of one extra gram paracetamol PRN per day can be accepted, provided that this is recorded in the patient’s medication sheet and does not occur more than three times in one week. If more paracetamol or other pain treatment is needed, the participant will (temporarily) stop study medication, but the measurements will continue (if possible), following the intention-to-treat principle. For participants who are unable to swallow tablets, the study medication will be administered by their usual way of medication intake.

### Randomisation, blinding and treatment allocation

After a screening visit by a research nurse, residents who are eligible for participation are randomised (1:1) into two groups (Fig. 1). Block randomisation (blocks of four) is used, generated by a computer random number generator in the pharmacy. Participants, informal caregivers, nursing staff, physicians, investigators and research nurses are blinded to treatment. The randomisation numbers combined with the allocated treatment arm (paracetamol/placebo or vice versa) are put in sealed envelopes and are under guidance of the researchers in case clarification of an allocation is needed. Only the study pharmacy of the Leiden University Medical Center knows which participant/code is allocated to which treatment arm. The researcher/research nurse shall only unblind the treatment allocation if this is relevant to the safety of the participant. In case of unblinding, the participant will quit study medication, but measurements will be continued (if possible) following the intention-to-treat principle. The same applies for participants quitting study medications for other reasons.

### Research questions and hypotheses

1. What is the effect of regularly scheduled administration of pain treatment with paracetamol on QoL in LTCF residents with advanced dementia, compared to placebo? We hypothesise that undiscovered and un(der)treated pain, causing moderate to poor QoL (as assessed by the QUALIDEM and the DS-DAT), might be resolved by scheduled pain treatment, and thereby, improve overall QoL.
2. What is the effect of regularly scheduled administration of pain treatment with paracetamol on neuropsychiatric symptoms, ADL function and care dependency in LTCF residents with advanced dementia, compared to placebo? We hypothesise that resolving possibly undiscovered pain in persons with dementia will lead to decreased neuropsychiatric symptoms, better ADL functioning and less care dependency.
3. What is the effect of regularly scheduled administration of pain treatment with paracetamol on pain and use of psychotropic and pain medication in LTCF residents with advanced dementia, compared to placebo? As hypothesised in research question 1, regularly scheduled administration of pain might resolve undiscovered pain. Moreover, by evaluating pain using a measurement tool developed for the observation of people with dementia, we hypothesise this will increase the attention that nurses pay to pain. Finally, we hypothesise that use of psychotropic and (extra) pain medication will decrease due to paracetamol treatment.

### Measurements

Prior to the study, the nursing staff receive training from the researchers on QoL and pain in persons with dementia, and receive instruction on how to observe pain. These skills can also be beneficial for patient care in LTCFs, even after this trial has ended.

During the study, demographic data are obtained by nursing staff. Severity of dementia is measured using the Reisberg GDS. This assessment tool rates the clinically identifiable stage of cognitive decline, with scores ranging from 1 (no cognitive decline) to 7 (very severe cognitive decline) [13]. Only persons with Reisberg GDS scores ≥5 at baseline will participate in this study. Table 1 represents the time schedule and an overview of the study measurements.

**Table 1:** Measurements and time schedule during the proposed study

	Visit 1 Screening	Visit 2 Baseline	Visit 3 6 weeks	Visit 4 7 weeks	Visit 5 13 weeks
Check inclusion/exclusion criteria	RN				
Demographic characteristics	Nurse				
Dementia (Reisberg GDS)	RN/nurse				
Comorbidity (FCI)		ECP			
Quality of life (QUALIDEM, DS-DAT)	Nurse	Nurse	Nurse	Nurse	Nurse
Neuropsychiatric symptoms (NPI-NH)		Nurse	Nurse	Nurse	Nurse
Functioning (Katz-15)		Nurse	Nurse	Nurse	Nurse
Care dependency (CDS)		Nurse	Nurse	Nurse	Nurse
Pain (MOBID-2)		Nurse	Nurse	Nurse	Nurse
(Co)-medication use		ECP	ECP	ECP	ECP
(Serious) Adverse Events ((S)AE’s)		ECP	ECP	ECP	ECP

*RN* Research nurse

*Nurse* Professional care giver in nursing home

*ECP* Elderly care physician

#### Primary outcome

##### Quality of life

At baseline and at follow-up, QoL will be measured with the QUALIDEM, which includes 37 items for observation by nurses on nine QoL domains (care relationships, positive affect, negative affect, restless tense, behaviour, positive self-image, social relations, social isolation, feeling at home, and having something to do) [17, 18]. This is a dementia-specific QoL measurement (initially developed for the Dutch population) and has satisfactory reliability and validity, also in other countries [17, 19] Only the 18 items that are also applicable for very severe dementia (GDS 7) will be included in this proposed study (as recommended by the authors in the QUALIDEM manual) [20]. These 18 items cover six QoL domains (care relationship, positive affect, negative affect, restless tense behaviour, social relations, and social isolation). The QUALIDEM is one of the few QoL instruments that focuses on the QoL domains that are considered important for persons with dementia, even in severe end-stage dementia, and is therefore a suitable instrument for the evaluation of QoL in persons with dementia [17, 19, 21, 22]. As it is recommended, we use the QUALIDEM together with the Discomfort Scale-Dementia of Alzheimer Type (DS-DAT; a measure to assess discomfort in dementia) to evaluate the influence of interventions and 24-h care on QoL in severe dementia [23]. The DS-DAT is a 9-item observational instrument that measures symptoms of discomfort of patients, regarding vocalisations, breathing, facial expression, and body movement. The Dutch version of the DS-DAT was found suitable to assess discomfort in nursing home residents that have severe dementia, and has proven to be valid and reliable [24–26].

#### Secondary outcomes

##### Neuropsychiatric symptoms

The Neuropsychiatric Inventory-Nursing Homes (NPI-NH) will be used to measure neuropsychiatric symptoms. The NPI-NH is based on a structured interview with an informant (in the proposed study: nursing staff) and consists of 10 domains of measuring behaviour (delusions, hallucinations, agitation/aggression, depression/dysphoria, anxiety, elation/euphoria, apathy/indifference, disinhibition, irritability/lability and aberrant motor behaviour), and two types of measuring neuro-vegetative changes (sleep and night-time behaviour disorders and appetite and eating disorders) [27]. Each symptom is valuated with frequency and severity scores; the sum of these 12 scores provides a total score, ranging from 0 (no symptoms at all) to 144 (all symptoms at every moment). The Dutch version of the NPI-NH has high interrater agreement, good construct validity, and can be scored objectively [28, 29]. In addition to the frequency and severity scores, we also use the Caregiver Distress Scale of the NPI, which assesses the level of caregiver (occupational) distress associated with the patient’s behavioural disturbances measured with the NPI, ranging from 0 (no distress) to 60 (very disruptive, major source of distress for staff) [30].

##### ADL functioning

The Katz ADL index is a reliable and valid instrument to measure ADL function [31]; it is also reliable and sensitive to change in persons with dementia [32]. The summary score of the Katz ADL index ranges from 0 (low function/fully dependent) to 15 (high function/fully independent) [33].The questionnaire is filled out by the nursing staff.

##### Care dependency

Care dependency is measured with the Care Dependency Scale (CDS), that assesses care dependency of institutionalised residents based on 15 items [34]. It is filled in by nursing staff and has satisfactory reliability and validity [35, 36]. The total score ranges from 15 (completely dependent on care) to 75 (almost independent of care).

##### Pain

The Mobilization-Observation-Behavior-Intensity-Dementia-2 (MOBID-2) pain scale is an observational pain tool for residents with advanced dementia [37, 38]. This assessment is based on observation of the resident’s immediate pain behaviour related to the musculo-skeletal system, doing standardised and guided movements during morning care [12]. The intensity of pain is rated by a nurse on a numerical rating scale, ranging from 0 (no pain) to 10 (pain as bad as it could possibly be) [39]. An overall score of ≥3 is indicative of a patient having clinically relevant pain [38, 40]. The MOBID-2 has good reliability and validity [41] and is responsive to change [12].

##### Psychotropic – And pain medication use

At baseline, a medication list is provided by the elderly care physician. In addition, copies of the drug registration forms are collected during the study period. Differences in use of psychotropic medication and/or use of (extra) pain medication during the study period are analysed as a secondary outcome measure.

##### Compliance

Compliance with study medication is registered on the drug registration form by the nurse each time paracetamol or placebo tablets are offered. Reasons for non-compliance are asked for and recorded. Additionally, a tablet count is performed after each intervention period. Non-compliance is defined as an adherence of < 90% as registered on the drug registration form (remaining tablet count of 24 or more, per period). Throughout the study, the nursing staff and physicians are asked to report (serious) adverse events and side effects of paracetamol/placebo use to the researchers on a structured questionnaire. At each study visit, we explicitly ask again for possible adverse events and side effects of paracetamol/placebo use that might not have been reported.

The study does not interfere in any way with standard care, diagnostics and treatment for persons with dementia.

### Sample size calculation

To detect an inter-individual difference of 10% on the QUALIDEM score with 80% power, and alpha 0.05, we calculated that a sample of 70 residents will be required. We assume an intra-individual standard deviation of 13 points, as derived from a previous study [42]. Estimating a dropout of 35% (mortality, loss to follow-up from other reasons, unwillingness to participate, existing pain, etc.) and invalid measurements of 5%, we plan to randomise 95 eligible patients.

### Statistical analysis

**Fig. 2:**
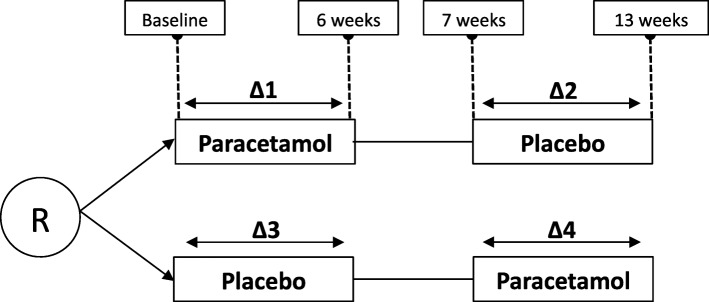
Time frames (Δ) used in the statistical analysis. R = randomisation. Δ1 = difference in outcomes between baseline and 6 weeks. Participant started with paracetamol. Δ2 = difference in outcomes between 7 weeks and 13 weeks. Participant started with paracetamol. Δ3 = difference in outcomes between baseline and 6 weeks. Participant started with placebo. Δ4 = difference in outcomes between 7 weeks and 13 weeks. Participant started with placebo

Outcomes will be compared in four different time points: at baseline, and after 6, 7 and 13 weeks.. First, we examine the degree of an order effect, i.e. whether there is a significant difference between Δ1-Δ2 and Δ4-Δ3 (see Fig. 2 for the Δ-time frames). Unpaired T tests will be used for normally distributed numerical data, one-way ANOVA tests for not normally distributed data, and Chi-squared tests for categorical data. Second, we look for the existence of a period effect, i.e. whether the difference between Δ2 and Δ4 is significantly different from the difference between Δ1 and Δ3. Paired t-tests are used for normally distributed numerical data, Wilcoxon signed-rank tests for no normal distributed data, and Chi-squared tests for categorical data. If no significant order effect or period effect is found, the mean/median outcomes of Δ1 and Δ4 are compared with the mean/median outcomes of Δ2 and Δ3, using paired t-tests for normally distributed numerical data, Wilcoxon signed-rank tests for not normally distributed data, and Chi-squared tests for categorical data. If any order or period effect is found, differences in QoL total and subdomain scores, neuropsychiatric symptoms, ADL functioning, care dependency, pain, and psychotropic and pain medication use are analysed with repeated (linear) mixed models, in which we adjust for order and period effect. Data will be presented quantitatively and processed using the SPSS package.

## Discussion

This randomised, double-blind, placebo-controlled crossover study will provide knowledge on the effectiveness of six weeks of regularly scheduled pain treatment with paracetamol on QoL in persons with dementia living in LTCFs with low QoL, compared to placebo. Persons with dementia are at high risk of experiencing negative consequences of pain, such as behavioural problems (agitation, apathy), decrease in ADL functioning, sleep problems and depression.

Paracetamol is reported to be beneficial for social interaction [6] and behavioural disturbances [4, 12]. Since paracetamol is known for its analgesic effect, use of paracetamol might decrease the negative consequences of pain and, thereby, improve QoL in persons with dementia.

Paracetamol rarely causes side effects, which can include headache or allergy. In case of long-term use of paracetamol (i.e. months or years), or doses exceeding the maximum recommended dose (3–4 g/day) side effects can include liver damage, kidney damage and blood abnormalities. Any inconvenience for the residents (taste of the tablets, swallowing more tablets than the person is used to in one day) or the nursing staff (measurements taking up time), will not outweigh the benefits described above.

The crossover design has the advantage that (on average) 75% fewer participants are needed to achieve the same satisfactory power as studies that have parallel groups without crossover of treatments. [43] Also, the characteristics of the participants of the two randomised treatment groups are the same at baseline (the same person receives paracetamol and placebo, only the order of administration differs); therefore, confounding is minimised when comparing the two treatments. The washout period minimises the carryover of effects from one treatment period to another. Also, randomising the participants minimises any potential period effect (i.e. the effect of time and/or seasonal changes on a person’s outcome) in the comparison of treatments [43].

A final benefit of this design is that, since participants receive both treatments, the results can be compared within one individual.

Within this vulnerable group of patients, there is always a risk of a high mortality rate and dropout during a study. Especially in a crossover study, this can be a problem, since participants are their own controls. Although this was taken into account when calculating the sample size, a high dropout could be a limitation of this study design (i.e. limiting the reliability of the results); although this also emphasises the difficulty of performing a study in this population. Another possible limitation is the percentage response of the consent that is requested from legal representatives by mail; we estimate that about 30% will be non-responders, and that about 50% of the responders will not allow the resident to participate in the study. Lastly, eligibility in this specific population is low, as (on average) ≥ 50% of persons with dementia already uses pain medication, the mortality rate between consent and screening can be high, and/or the residents might meet one or more of the exclusion criteria (e.g.severe psychiatric disease, low weight). Low eligibility was also shown in the DEP.PAIN.DEM study, in which 2200 patients had to be contacted in order to include just over 160 patients [5]. Based on ethical considerations, we will not specifically ask the elderly care physician to stop pain treatment in a patient in order to meet our eligibility criteria.

If the results of this proposed trial show that six weeks of paracetamol improves the QoL of persons with dementia and a low QoL, we need to be aware of the potential consequences for daily practice in long-term care. The message would certainly *not* be to give every person with dementia daily paracetamol. Recent increases in analgesic use in persons with dementia, especially in Scandinavian countries, have raised the question as to whether this was based on sound individual evaluation and monitoring, or a reaction to reports on under-treatment [44]. It is likely (and has also been reported), that the effect of psychosocial interventions such as pleasant activities, exercise, as well as reminiscence and music therapy, is small or even absent if there is unnoticed or poorly managed pain [45]. Therefore, the message would be that nursing staff should regularly measure QoL in dementia, and that paracetamol for six weeks may be the first intervention to improve QoL of persons with advanced dementia and a low QoL.

## Abbreviations

ADL: Activities of daily living
CDS: Care dependency scale
GDS: Global deterioration scale
LTCF: Long-term care facility
MOBID-2: Mobilization-Observation-Behavior-Intensity-Dementia-2
NPI-NH: Neuro Psychiatric Inventory – Nursing Home version
PRN: pro re nata (‘as needed’)
QoL: Quality of life
Q-PID: Quality of life and Paracetamol In advanced Dementia
RN: Research nurse
